# Positive selection in the adhesion domain of *Mus* sperm *Adam* genes through gene duplications and function-driven gene complex formations

**DOI:** 10.1186/1471-2148-13-217

**Published:** 2013-09-30

**Authors:** Phil Grayson, Alberto Civetta

**Affiliations:** 1 Department of Biology, University of Winnipeg, Winnipeg, Canada; 2 Current Address: Department of Organismic and Evolutionary Biology, Harvard University, Cambridge, USA

**Keywords:** Positive selection, *Adam* genes, Sperm, Neofunctionalization, Rodents

## Abstract

**Background:**

Sperm and testes-expressed *Adam* genes have been shown to undergo bouts of positive selection in mammals. Despite the pervasiveness of positive selection signals, it is unclear what has driven such selective bouts. The fact that only sperm surface *Adam* genes show signals of positive selection within their adhesion domain has led to speculation that selection might be driven by species-specific adaptations to fertilization or sperm competition. Alternatively, duplications and neofunctionalization of *Adam* sperm surface genes, particularly as it is now understood in rodents, might have contributed to an acceleration of evolutionary rates and possibly adaptive diversification.

**Results:**

Here we sequenced and conducted tests of selection within the adhesion domain of sixteen known sperm-surface *Adam* genes among five species of the *Mus* genus. We find evidence of positive selection associated with all six *Adam* genes known to interact to form functional complexes on *Mus* sperm. A subset of these complex-forming sperm genes also displayed accelerated branch evolution with *Adam5* evolving under positive selection. In contrast to our previous findings in primates, selective bouts within *Mus* sperm *Adams* showed no associations to proxies of sperm competition. Expanded phylogenetic analysis including sequence data from other placental mammals allowed us to uncover ancient and recent episodes of adaptive evolution.

**Conclusions:**

The prevailing signals of rapid divergence and positive selection detected within the adhesion domain of interacting sperm *Adams* is driven by duplications and potential neofunctionalizations that are in some cases ancient (*Adams* 2, 3 and 5) or more recent (*Adams* 1b, 4b and 6).

## Background

The majority of protein coding genes analyzed through molecular evolutionary studies have been found to evolve under purifying selection, but genes that function in perception, immunity and reproduction are often fast-evolving exceptions to this rule [1-3]. Reproductive genes, such as those that code for species-specific fertilization proteins, male accessory gland proteins, and sperm proteins have been shown to exhibit rapid evolution in taxa as diverse as invertebrates, mammals and plants [4-9].

The ADAM (A Disintegrin And Metalloprotease) gene family contains at least 35 members in mammals, with more than half known to be testes-expressed. The analysis of ADAM family evolution among mammals has found faster divergence of genes expressed in testes with evidence of positive selection at codon sites within the adhesion domain of sperm surface genes [10-13]. This localization of adaptive selection in *Adam* genes led us to hypothesize an important role in sperm-egg interactions with positive selection possibly driven by sexual selection. Thus far, very few studies have successfully linked bouts of positive selection at sperm surface genes to differences in mating systems, testes masses or other proxies of sexual selection [14].

Sexual selection is likely to have driven positive selection within the adhesion domain of sperm *Adam* genes in rodents. In the *Mus* genus, sperm competition favours larger numbers of sperm and has resulted in marked differences in relative testes size between different species, with *Mus spicilegus* having the largest relative testes mass (RTM) and relative testes weight (RTW) [15-17]. Moreover, knockout mice for ADAM2 and ADAM3 show drastic decreases in sperm aggregation, a trait that has been suggested to confer sperm with competitive advantages [18-20].

Alternatively, localized signals of positive selection within the adhesion domain of *Adam* genes could result from species-specific adaptations to fertilization. There are sixteen *Adam* sperm surface genes in mice, with twelve known to be localized on mature sperm and three (*Adams* 1, 2 and 3) being directly linked to sperm migration and sperm-egg adhesion and fusion. ADAM3 knockouts appear to have the most severe effect on reproductive fitness, resulting in infertile males due to deficiencies in sperm-zona pellucida (ZP) interactions, and more importantly, sperm migration into the oviduct [21-23]. ADAM2 knockouts also significantly affect reproductive success. *In vivo*, ADAM2 null mice have a fertility rate 50 times lower than the wild-type. This drop in fertility once again does not appear to be the result of a single process, but is instead a combination of deficiencies in sperm-egg fusion, sperm-egg binding, sperm-ZP binding and sperm migration [24]. ADAM1a knockouts result in sperm unable to migrate to the egg; *in vivo* the knockout produces an infertile phenotype but *in vitro*, sperm are able to fertilize eggs. ADAM1b knockouts appear to produce normal sperm but affect the levels of ADAM2 on mature sperm [25,26]. Interestingly, six of the sperm surface genes (*Adam*s 1 to 6) assemble into functional complexes. Currently, there is evidence for three sperm-specific complexes (ADAM2-ADAM3-ADAM4, ADAM2-ADAM3-ADAM5, and ADAM2-ADAM3-ADAM6), two testes-specific complexes (ADAM1a-ADAM2, and ADAM2-ADAM3), and one complex common to both (ADAM1b-ADAM2) [27]. All complexes require at least ADAM2 and/or ADAM3, if not both, and their interactions appear to be central for a variety of sperm functional adaptations to fertility in mice.

One final consideration is that while the inclusion of a wide range of species in prior phylogenetic studies of *Adam* genes has served to resolve some aspects of the history of the gene family, the use of very distant species makes the proper identification of selective pressures more difficult [12,28,29]. This is because bouts of selection can be localized to specific clades or even branches within the phylogeny, causing a failure to detect a signal of selection when a wide range of species are included in the analysis [12,30]. Similarly, analyses utilizing wide ranges of ancient paralogs that cluster within a phylogenetic clade can fail to detect positive selection that is gene-specific [29].

Here we present novel sequence data for all sperm-expressed members of the ADAM family in *Mus*. Sequences were utilized to conduct tests of selection within the adhesion domain of sixteen known sperm surface genes among five species of the *Mus* genus. We find evidence of positive selection associated with all six sperm surface *Adam* genes involved in interacting complexes within the *Mus* phylogeny, and see accelerated branch evolution for some. The selective bouts showed no associations to proxies of sperm competition. We expanded the phylogenetic analysis to include species within the Glires clade and from the superorder Laurasiatheria and found positive selection across groups (ancient) for *Adams* 2, 3 and 5 and more recent localized bouts of selection for *Adams* 1b, 4b and 6 in *Mus*/Glires.

## Methods

### DNA samples and sequencing

Genomic DNA was obtained from the Jackson Laboratory (http://www.jax.org) for *M. musculus musculus*, *M. m. domesticus*, *M. spretus*, *M. spicilegus* and *M. caroli* (Strain Names: SKIVE/Ei, LEWES/Ei, SPRET/Ei, PANCEVO/Ei and *Mus caroli*/Ei respectively). Primers were designed for the adhesion domain of all known *Mus* sperm *Adams* (1a, 1b, 2, 3, 4a, 4b, 5, 6a, 6b, 7, 18, 24, 26a, 26b, 30 and 32) using Primer3 (http://frodo.wi.mit.edu/) and published *M. m. domesticus* sequence data (Additional file 1: Table S1). The adhesion domain was identified for each gene using The Motif Scan Server’s Prosite options (http://myhits.isb-sib.ch/cgi-bin/motif_scan). Polymerase chain reaction (PCR) amplification of adhesion domain exons was carried out with Phusion High-Fidelity DNA Polymerase (Thermo Scientific) in 12.5 and 25.0 μl reactions using the following general conditions: initial denaturation for 30 s (98°C) was followed by 35 cycles of denaturation for 10 s (98°C), annealing for 30 s (temperature gradient), and extension for 9 s (72°C). A final extension step was carried out for 5 min (72°C). Optimization of most primer pairs resulted in a species-specific temperature gradient for the annealing step that ranged from 55-70°C (see Additional file 1: Table S2 for optimized primer conditions).

Optimized PCR products were Sanger sequenced at the SickKids TCAG DNA Sequencing Facilities in Toronto, Canada. Products were sequenced using both the forward and reverse primers and a consensus sequence for each species was constructed using ClustalW in Mega 5.2 with visual inspection and manual adjustments [31,32]. For some *Adam* genes, *M. spretus* sequences were retrieved from the SNP database (http://www.sanger.ac.uk/cgi-bin/modelorgs/mousegenomes/snps.pl). All our sequence data has been deposited in Genbank under accession numbers KF144343 to KF144410.

### Phylogenetic reconstruction

The Basic Local Alignment Search Tool (BLAST) at NCBI (http://blast.ncbi.nlm.nih.gov/Blast.cgi) was used to query sequence data against the mouse genome to ensure that primers had correctly isolated targeted *Adam* exons. To further enhance confidence in *Mus* species sequencing data, and to confirm orthology among different *Adam* genes, we included our sequences within a larger mammalian phylogeny. We collected *Adam* gene sequence data for 98 additional mammals from NCBI and Ensembl (See Additional file 1: Table S1 for accession numbers) and aligned the adhesion domain using ClustalW in Mega5.2. The ProtTest 2.4 Server was utilized to determine the best model of protein evolution for phylogenetic reconstruction [33]. The phylogeny was built in Mega 5.2 using Maximum Likelihood and the reliability of the tree branching was assessed using 1,000 bootstrap replicates [34]. This alignment and phylogeny has been deposited to treeBASE as indicated in the Availability of supporting data section.

### Phylogenetic branch and site tests of selection

Alignments were analyzed using different models within the codeml program of PAML v. 4.7 using an unrooted *Mus* phylogeny [35]. The likelihoods of the one ratio model, with ω estimated or fixed at 1.0, and that of the free-ratio model were compared. We also used the two-ratio and the branch-site models to test for differential evolutionary rates and selection linked to proxies of sexual selection. The tests were done by flagging the ancestral branch and the branches leading to the two species with larger relative testes measurements (*M. spicilegus* and *M. spretus*). Two measures of testes size are commonly used in the literature, relative testes weight (RTW) and relative testes mass (RTM). Depending on the scale used, there is a 4 to 5-fold difference in relative testes size for the *Mus* species examined in this work. *M. spicilegus* boasts the largest values (RTW = 0.030, RTM = 1.682) followed by *M. spretus* (RTW = 0.017, RWM = 1.072). The remaining species utilized in this study have the following values: *M. m. musculus* (RTW = 0.006, RTM = 0.411), *M. m. domesticus* (RTW = 0.008, RTM = 0.506) and *M. caroli* (RTW =0.007, RTM = 0.434) [15-17].

The likelihoods of the site models M8 and M8a were calculated to identify, within the *Mus* phylogeny, genes and sites that had experienced positive selection. These models allow ω to vary among codon sites; model M8 assumes that positive selection might occur and ω values can exceed 1 while the null model, M8a, fixes ω at 1. For genes showing evidence of positive selection within the *Mus* genus, the M8-M8a tests of selection were conducted for species of the Glires clade (Rodentia and Lagomorpha) and the superorder Laurasiatheria. This approach was used to determine whether bouts of positive selection were restricted to *Mus* and/or Glires or more ancestrally shared with other placental mammals. The Bayes Empirical Bayes (BEB) method was conducted in conjunction with model M8 to identify specific amino acid sites experiencing bouts of adaptive evolution [36].

### Additional tests of selection and functional divergence

Tests of site-specific functional divergence were conducted on complex-forming paralogous *Mus* sperm *Adams* (1a/1b, 4a/4b, 6a/6b) using the GU99 method within DIVERGE v. 1.04 [37,38]. This analysis identifies gene pairs evolving under functional divergence, where one duplicate is highly conserved while the other evolves in a highly variable manner [39]. TreeSAAP v. 3.2, a program that examines amino acid substitution by measuring selection’s effect on 31 different amino acid properties, was utilized within the expanded phylogenies (Glires and Laurasiatheria) to identify sites of positive selection for *Adams* 1, 2, 3, 4 and 5 [40]. These two analyses provide amino acid sites believed to be positively selected or integral for functional divergence. Sites identified through TreeSAAP were utilized to validate or reject those from the PAML BEB results, while DIVERGE assessed if amino acids identified as evolving under positive selection by BEB in PAML contributed to functional divergence between paralogous *Adam* genes.

## Results

**Figure 1:**
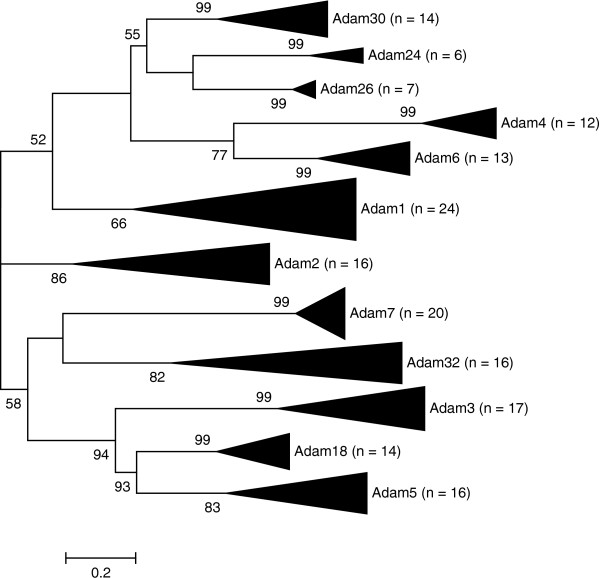
**Sperm *Adam *molecular phylogeny supporting *Adam *sequence orthology.** The WAG model of protein evolution with G, the gamma distribution shape parameter, and I, invariant sites, was selected for the phylogenetic reconstruction. The model was selected based on its likelihood and AIC (akaike information criterion) value (Additional file 1: Table S3).

Our phylogenetic reconstruction supported the overall orthology of mammalian *Adam* genes used in this study (Figure 1 and Additional file 1: Table S3). The nested branch models of evolution showed that in most cases a model that allows for different ω estimates per tree branch was not necessarily a better fit to the data than a model assuming a single estimated ω for all branches in the *Mus* tree (Additional file 1: Table S4). For *Adams* 1a, 4a, 6a, 6b, 7 and 30, an average branch ω significantly lower than one (purifying selection) was a better fit to the data than an average branch ω of one. *Adam4b* also showed a significant result in support of purifying selection at a 10% level of significance (FDR corrected *P* < 0.1), whereas *Adam5* was found to be evolving under positive selection throughout the phylogeny (Table 1 and Additional file 1: Table S4). Those *Adams* not listed above had average branch ω values non-significantly different from one, indicating rapid branch evolution due to relaxed selection (Table 1). It is noteworthy that several genes (*Adam*s 1b, 2, 3, 4 and 5) that interact to form functional complexes on mature mouse sperm are among the relaxed/positively selected genes with large average ω branch values (Table 1).

**Table 1:** **Branch and site tests of selection in *Mus***

***Gene***	***l***	***n***	***2Δℓ M0N0***	***ω***	***Branch***	***2Δℓ***	***p***_***1***_***; ω (M8)***	***Site***
			***(ω vs ω = 1.0)***		***Selection***	***(M8-M8a)***		***Selection***
*Adam1a*	321	5	**9.78**	0.16	Purifying	0		Purifying
*Adam1b*	321	5	0.03	0.91	Relaxed	3.89	0.07; 9.10	Positive
*Adam1*	321	10	**63.11**	0.21	Purifying	1.94		Purifying
*Adam2*	333	5	0.2	0.78	Relaxed	**9.77**	0.11; 12.87	Positive
*Adam3*	339	5	0.04	0.89	Relaxed	4.08	0.13; 8.92	Positive
*Adam4a*	327	5	**6.18**	0.22	Purifying	0		Purifying
*Adam4b*	327	5	3.25	4.69	Purifying	**15.36**	0.04; 38.74	Positive
*Adam4*	327	10	0.8	1.39	Relaxed	**49.84**	0.07; 23.54	Positive
*Adam5*	273	5	**4.36**	3.78	Positive	**24.38**	0.20; 30.03	Positive
*Adam6a*	327	5	**9.75**	0.21	Purifying	2.02		Purifying
*Adam6b*	327	5	**9.74**	0.17	Purifying	0.65		Purifying
*Adam6*	327	10	**11.67**	0.32	Purifying	**8.15**	0.08; 5.69	Positive
*Adam7*	267	5	**11.03**	0.15	Purifying	0		Purifying
*Adam18*	339	5	1.96	0.46	Relaxed	2.21		Purifying
*Adam24*	330	5	0.06	1.15	Relaxed	**7.64**	0.06; 19.11	Positive
*Adam26a*	327	4	0.07	0.87	Relaxed	1.53		Purifying
*Adam26b*	327	4	1.35	0.5	Relaxed	0.11		Purifying
*Adam26*	327	7	0.24	0.78	Relaxed	4.03	0.29; 3.63	Positive
*Adam30*	327	5	**7.95**	0.14	Purifying	0		Purifying
*Adam32*	285	5	1.91	0.46	Relaxed	0.01		Purifying

n = number of species included; l = length of adhesion domain analyzed (bp); 2Δℓ = the difference in likelihood estimates from different models of selection; ω= d_N_/d_S_ per codon for sites under positive selection; p_1_ = proportion of codon sites under positive selection. Test statistics (*2Δℓ*) are bolded (FDR corrected *P* < 0.05) or underlined (FDR corrected *P* < 0.10) to denote statistical significance.

While there was evidence of an overall constancy of ω ratios across all branches in the *Mus* phylogeny, it is possible that localized bouts of sexual selection within branches might have been lost in such a test. Therefore we tested branches leading to species with larger testes, as a proxy of sperm competition, by estimating the likelihood of a model with estimated ω along the foreground and background branches against a model with the foreground branch ω fixed at 1.0. Down the *M. spicilegus* and *M. spretus* foreground lineages, an estimated ω < 1.0 was only a better model for *Adams* 4a and 7. Results from the branch-site model within PAML also indicated that codon sites specific to *M. spicilegus* and/or *M. spretus* were not evolving more quickly under the influence of sexual selective pressures. Thus, we found evidence of purifying selection for two *Adam* genes and no indication of sexual selection at the branch ancestral to the species with largest RTW and RTM or at the branch leading to *M. spicilegus* or *M. spretus* individually using two separate tests (Additional file 1: Tables S4 and S5).

Despite the apparent constancy of evolutionary rates within branches of the *Mus* phylogeny, it is possible that some codon sites might have been influenced by bouts of positive selection. Likelihood ratio tests comparing PAML’s site models (M8 and M8a) were conducted on each of the 16 known *Mus* sperm *Adams* (as listed in the Methods section). The results indicate that positive selection has driven the evolution of codon sites within *Adams* 2, 4b, 5 and 24 (FDR corrected *P* < 0.05), with *Adam*s 1b, 3 and 26 also showing evidence of positive selection (FDR corrected *P* < 0.1) (Table 1). When paralogous gene pairs were examined together, positive selection was also found to influence the evolution of *Adams* 4, 6 and 26 (Table 1). We used DIVERGE analysis on *Adams* 1, 4 and 6 to assess the potential role of positively selected sites on functional divergence between gene paralogs. For *Adam1*, seven out of nine positively selected sites were supported as contributors to functional divergence between *Adams* 1a and 1b (Additional file 2: Figure S1). Nine out of eleven positively selected sites were supported as contributors to functional divergence between *Adams* 4a and 4b (Additional file 2: Figure S2)*. Adams* 6a and 6b did not show positive selection by themselves but they did when analyzed together (Table 1). None of the positively selected sites detected using PAML were found to contribute to functional divergence between these paralogs (Additional file 2: Figure S3).

Expanded phylogenies for Glires (including our *Mus* sequences) and Laurasiatheria, were produced for all complex-forming sperm *Adams* to examine whether the signals of positive selection identified for *Mus* were clade specific, shared with Rodentia and Lagomorpha (Glires), or ancient and shared with other placental mammals (Laurasiatheria). PAML analyses suggested that *Adams* 2, 3 and 5 displayed site-specific evidence of positive selection throughout the Glires and Laurasitheria, indicating that these signals are not *Mus*-specific. In contrast to these results, clade-specific bouts of selection appear to be present for *Adams* 1, 4 and 6. Positive selection of *Adam1* in *Mus* is restricted to *Adam1b* (Table 1), a pattern mirrored by the paralogs in Glires, with no evidence of positive selection within Laurasiatheria (Table 2). There were not enough sequences available for *Adams* 4 and 6 to run the analysis outside Glires, but a signal of positive selection was found for *Adam4b* but not *Adam4a* in *Mus* (Table 1), so the situation could be similar to what was seen for *Adam1*. *Adam6* was only positively selected when both paralogs (6a and 6b) were pooled together in the analysis (Table 1), and the signal was lost when other species of Glires were included, suggesting that positive selection is localized in *Mus*. The majority of positively selected sites detected by PAML’s BEB analysis within Glires and Laurasiatheria were validated using TreeSAAP (Table 2).

**Table 2:** **Sperm *Adam *site selection for expanded species groups showing clade localization of selective bouts**

***Gene***	***Group***	***n***	***p1; ω (M8)***	***2Δℓ (M8-M8a)***	***BEB sites (P > 95%)***	***TreeSAAP sites (BEB)***
*Adam1a*	Glires	9		1.51		
*Adam1b*	Glires	9	0.24; 1.93	**4.56**	2	30 (2)
*Adam1*	Glires	18	0.16; 2.05	**10.89**	4	59 (4)
	Laurasiatheria	6		2.44		
*Adam2*	Glires	9	0.02; 21.73	**11.34**	2	38 (2)
	Laurasiatheria	7	0.33; 2.54	**22.71**	16	55 (15)
*Adam3*	Glires	9	0.03; 8.44	**5.07**	1	27 (1)
	Laurasiatheria	8	0.13; 2.64	**10.05**	1	48 (1)
*Adam4*	Glires	12	0.31; 3.98	**24.71**	11	20 (9)
	Laurasiatheria	2	Not Run			
*Adam5*	Glires	8	0.05; 21.51	**23.32**	4	20 (3)
	Laurasiatheria	8	0.16; 3.55	**24.09**	9	37 (9)
*Adam6*	Glires	13		0.6		
	Laurasiatheria	3	Not Run			

n, 2Δℓ, ω, and p_1_ are utilized as in Table 1. BEB Sites are the number of sites identified as being under positive selection using model M8 in PAML and a posterior probability threshold higher than 95%. TreeSAAP Sites are the number of positively selected sites as identified by TreeSAAP with the number of sites shared with PAML in brackets. Significance of the *2Δℓ* test statistics is depicted as in Table 1.

## Discussion

The lack of evidence of linkages between proxies of sexual selection and adaptive evolution in *Mus* is in contrast to prior evidence we have found of sexual selection in primates for *Adam2* and *Adam18 *[12]. This result highlights the importance of testing selection within specific groups or clades before making generalizations about sexual selection, as selective bouts can be lineage-specific and thus lost in phylogenetic analysis that include widely diverged species [41]. In fact, based on evidence on the potential role of sperm aggregation in sperm competition, it is possible that sexual selection might drive the evolution of some sperm *Adam* genes in different genera such as *Apodemus* (common wood mouse) and *Peromyscus* (deer mice) [18,19]. The different results observed between *Adam* genes in primates [12] and species of the *Mus* genus could also be explained if any relationship between proxies of sperm competition and positive selection is driven by transitions from monandry to polyandry.

Results for the site model (M8 vs. M8a) indicate that positive selection has driven the evolution of codon sites within *Adams* 1b, 2, 3, 4b, 5, 6, 24 and 26. Six of these sperm surface genes (*Adam*s 1–6) interact in forming functional complexes, and an interesting divide exists within these positively selected genes in *Mus*. As examined by Huxley-Jones and colleagues, *Adams* 2, 3 and 5 are closely related members of clade B [28]. They are all large, single-copy, multi-exon genes that display positive selection throughout the Glires clade and the superorder Laurasiatheria, indicating that these selective bouts are ancient and not *Mus*-specific. *Adams* 1, 4 and 6 belong to the more recently derived clade C [28]. They are all small, single-exon genes that are thought to have arisen through retrotransposition of spliced *Adam* mRNA into an ancestral genome [27]. In rodents, paralogous members of clade C are likely the result of even more recent tandem duplications, as they are situated adjacent to their duplicates on chromosomes 5 (*Adam1*) and 12 (*Adams* 4 and 6). In agreement with a recent origin, we found positive selection for clade C *Adams* within *Mus* affecting either a single member of the gene pair or when both paralogs were analyzed together. The pairing of purifying and positive selection seen for *Adams* 1a and 1b alongside differences in protein localization and function is in support of the classical model of neofunctionalization [25,26,42]. *Adam* 4 paralogs show the same pattern of purifying and positive selection, which is also strongly suggestive of neofunctionalization, but nothing is yet known about the duplicate functions or protein localization. The fact that most codon sites identified to be under positive selection in *Adam1b* and *Adam4b* in *Mus* are also found to contribute to functional divergence between paralogs provides further support to the hypothesis of neofunctionalization of both *Adams* 1 and 4. *Adam1* showed no evidence of selection within Laurasiatheria and the *Adam6* signal was lost when other species of Glires were included, suggesting that positive selection within these clade C *Adams* is specific to *Mus*. Interestingly, *Adam6* showed no evidence of functional divergence between paralogs which might suggest a more recent duplication event or weaker selective pressures acting on this gene.

It is yet unclear what drives positive selection at *Adam*s 24 and 26 (Table 1). *Adam26* is another member of clade C, and although little is known about its function, the positive selection signal within its adhesion domain is similar to that of *Adam6*, thus likely driven by a relatively recent duplication event. *Adam24*^*-/-*^ mice have been functionally assessed previously. They show a 50% drop in fertility with an increased number of sperm fusing to each egg, supporting the hypothesis that ADAM24 functions as an important sperm component to block polyspermy [43]. It is therefore possible that the adaptive evolution of *Mus Adam24* might be linked to species-specific adaptation to trigger such blocks.

## Conclusions

We have tested positive selection within the adhesion domain of all sperm-expressed members of the *Mus Adam* gene family and have identified an important role played by duplications and neofunctionalization. We also established that these bouts are not driven by selection linked to sperm competitive pressures. This stands in contrast to our previous findings in primates and highlights the importance of phylogenetically testing bouts of selection within specific species groups [12]. An expansion of the phylogenetic analysis beyond *Mus* highlights a dichotomy in the mode of evolution of the adhesion domain of sperm surface *Adam* genes driven by a combination of ancient (*Adams* 2, 3 and 5) and more recent (*Adams* 1b, 4b and 6) neofunctionalization of complex forming sperm proteins.

## Availability of supporting data

The data set supporting the results of this article is available from the TreeBase repository, http://purl.org/phylo/treebase/phylows/study/TB2:S14416. Sequencing data supporting the results of this study is available from NCBI under the following accession numbers: KF144343 to KF144410.

## Competing interests

Both authors declare that they have no competing interests.

## Authors’ contributions

AC conceived of the study. PG carried out molecular genetic studies and bioinformatics. PG and AC analyzed the final dataset and wrote the manuscript. Both authors read and approved the final manuscript.

## Supplementary Material

Additional file 1 **Tables S1, S2, S3, S4 and S5.** Accession numbers for species included in expanded phylogeny, primer conditions, ProtTest output, nested model (M0, M1, M2) likelihood ratio test results from PAML, and branch site model likelihood ratio results from PAML. Click here for file

Additional file 2 **Figures S1, S2 and S3.** Alignments of positively selected and functionally divergent sites as identified by Bayes Empirical Bayes in PAML and GU99 in Diverge for paralogous complex-forming Adam genes (1, 4, and 6). Click here for file
