# Supplementary material for: Positive selection in the adhesion domain of Mus sperm Adam genes through gene duplications and function-driven gene complex formations
Source: BMC Evol Biol. 2013 Sep 30;13:217. doi: 10.1186/1471-2148-13-217 (PMC3849967; doi:10.1186/1471-2148-13-217)
Supplement: Additional file 2 — Figures S1, S2 and S3. Alignments of positively selected and functionally divergent sites as identified by Bayes Empirical Bayes in PAML and GU99 in Diverge for paralogous complex-forming Adam genes (1, 4, and 6). [file 1471-2148-13-217-S2.pdf]

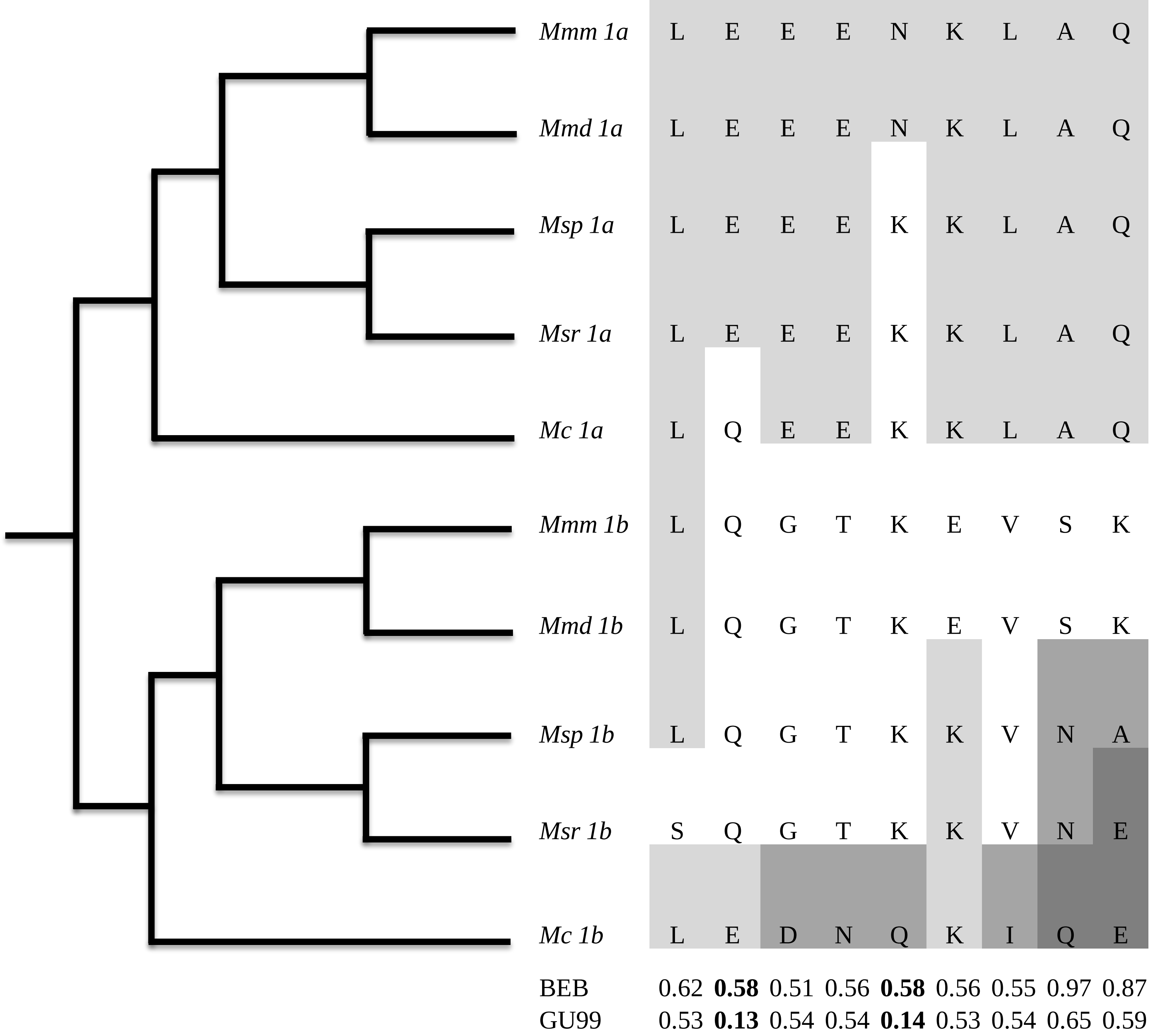

Supplementary Figure 1. Site-specific posterior probabilities of positive selection and functional divergence for *Adam1b* as identified by Bayes Empirical Bayes (BEB) using M8 in PAML and GU99 in Diverge. Positively selected sites not identified as contributors to functional divergence between paralogs are bolded. *Mmm* = *M. m. musculus*, *Mmd* = *M. m. domesticus*, *Msp* = *M. spicilegus*, *Msr* = *M. spretus*, *Mc* = *M. caroli*.

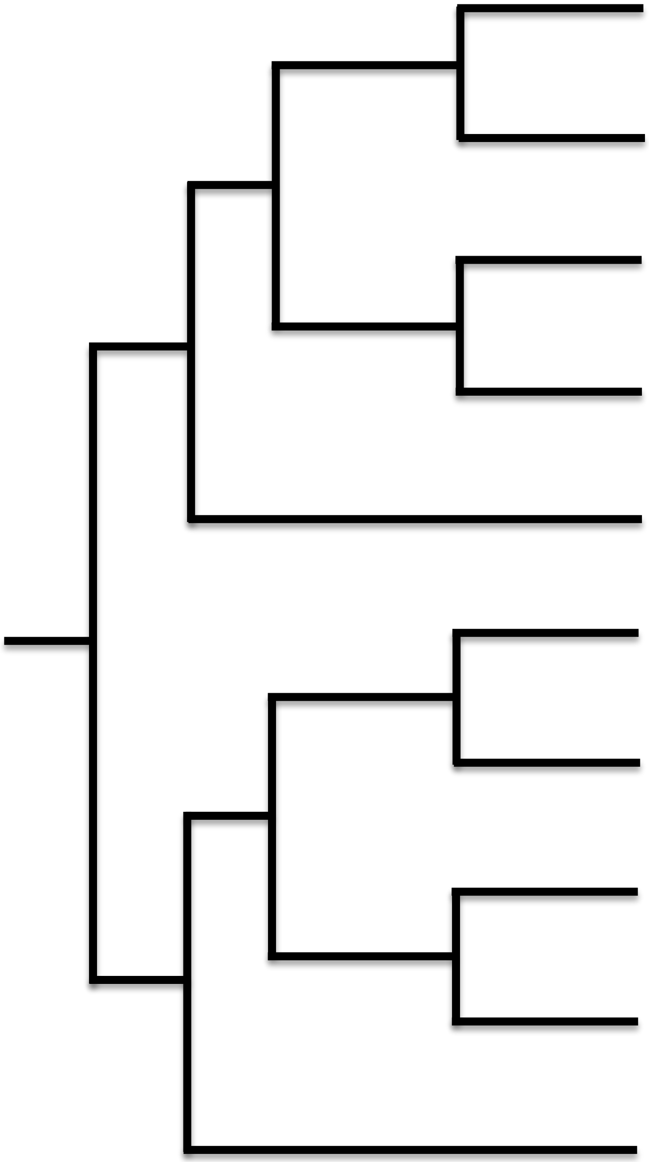

Mmm 4a	R	A	K	V	N	Q	A	A	L	A	S
Mmd 4a	R	A	K	V	N	Q	A	A	L	A	S
Msp 4a	R	A	K	V	N	Q	A	A	L	V	S
Msr 4a	R	A	K	V	N	Q	A	A	L	V	S
Mc 4a	K	A	K	V	N	Q	A	V	R	V	S
Mmm 4b	I	G	K	V	D	Q	N	R	L	A	T
Mmd 4b	I	G	S	V	D	Q	N	K	L	A	T
Msp 4b	I	G	S	V	D	Q	N	K	L	A	T
Msr 4b	I	G	S	V	D	Q	N	K	L	A	T
Mc 4b	I	A	T	L	D	P	N	T	L	V	S
BEB	1.00	0.94	1.00	0.63	0.94	0.66	1.00	**1.00**	0.68	**1.00**	0.69
GU99	0.95	0.73	0.84	0.73	0.64	0.73	0.65	**0.10**	0.95	**0.22**	0.73

Supplementary Figure 2. Site-specific posterior probabilities of positive selection and functional divergence for *Adam4* as identified by Bayes Empirical Bayes (BEB) using M8 in PAML and GU99 in Diverge. Positively selected sites not identified as contributors to functional divergence between paralogs are bolded. *Mmm* = *M. m. musculus*, *Mmd* = *M. m. domesticus*, *Msp* = *M. spicilegus*, *Msr* = *M. spretus*, *Mc* = *M. caroli*.

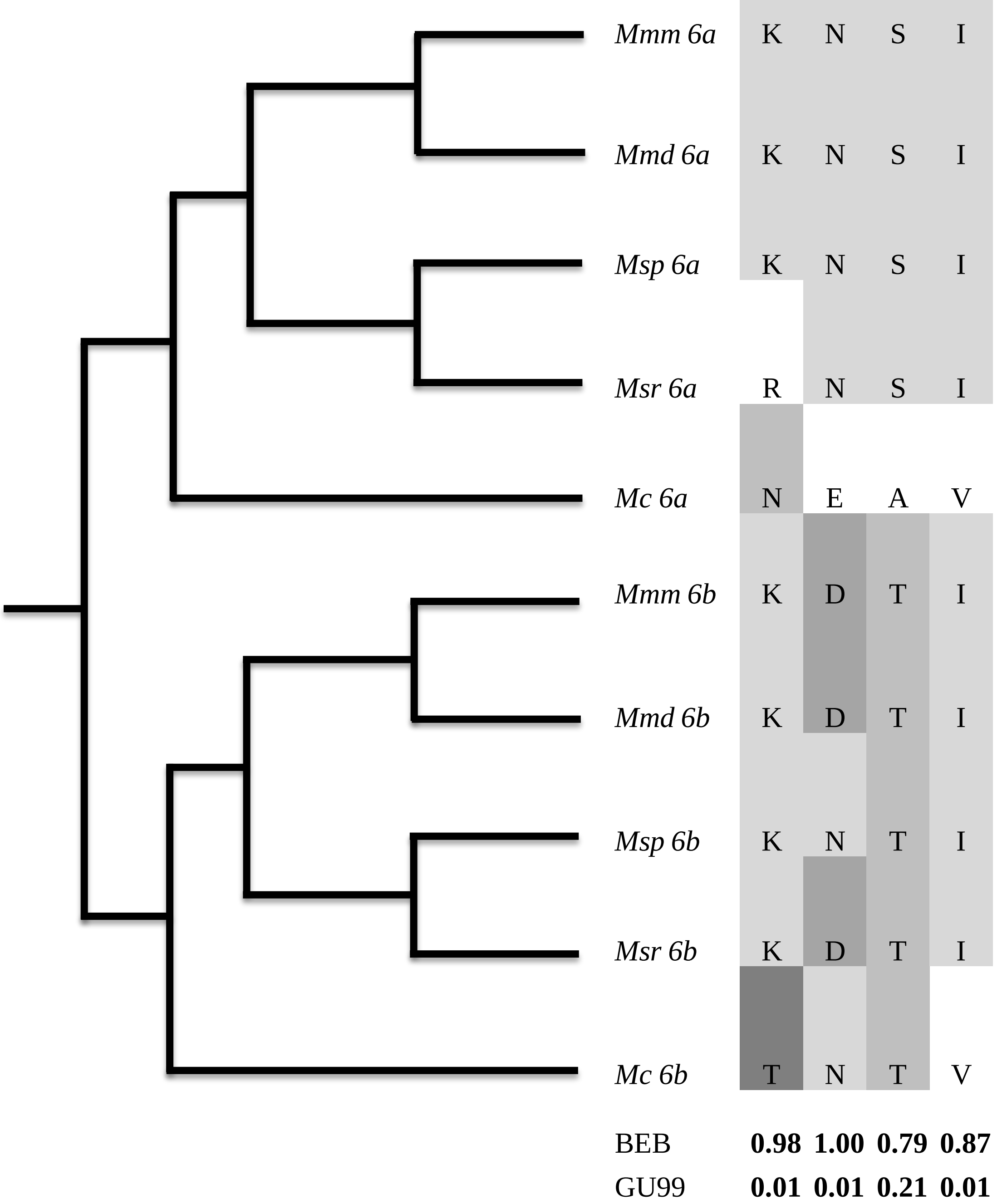

Supplementary Figure 3. Site-specific posterior probabilities of positive selection and functional divergence for *Adam6* as identified by Bayes Empirical Bayes (BEB) using M8 in PAML and GU99 in Diverge. Positively selected sites not identified as contributors to functional divergence between paralogs are bolded. *Mmm = M. m. musculus*, *Mmd = M. m. domesticus*, *Msp = M. spicilegus*, *Msr = M. spretus*, *Mc = M. caroli*.
